# The Quality of Practice Guidelines for Melanoma: A Methodologic Appraisal with the AGREE II and AGREE-REX Instruments

**DOI:** 10.3390/cancers12061613

**Published:** 2020-06-18

**Authors:** Theresa Steeb, Anja Wessely, Konstantin Drexler, Martin Salzmann, Frédéric Toussaint, Lucie Heinzerling, Markus Reinholz, Carola Berking, Markus V. Heppt

**Affiliations:** 1 Department of Dermatology, University Hospital Erlangen, Friedrich-Alexander-University Erlangen-Nürnberg (FAU), 91054 Erlangen, Germany; theresa.steeb@uk-erlangen.de (T.S.); anja.wessely@uk-erlangen.de (A.W.); Frederic.Toussaint@uk-erlangen.de (F.T.); lucie.heinzerling@uk-erlangen.de (L.H.); carola.berking@uk-erlangen.de (C.B.); 2 Comprehensive Cancer Center Erlangen-European Metropolitan Area of Nürnberg (CCC ER-EMN), 91054 Erlangen, Germany; 3 Department of Dermatology, University Hospital Regensburg, 93053 Regensburg, Germany; Konstantin.Drexler@klinik.uni-regensburg.de; 4 Department of Dermatology, University Hospital Heidelberg, 69120 Heidelberg, Germany; Martin.Salzmann@med.uni-heidelberg.de; 5 Department of Dermatology and Allergy, University Hospital, Ludwig-Maximilian University, 80337 Munich, Germany; markus.reinholz@med.uni-muenchen.de

**Keywords:** cutaneous melanoma, AGREE, level of evidence, melanoma treatment, practice guideline

## Abstract

Multiple guidelines on cutaneous melanoma (CM) are available from several consortia and countries. To provide up-to-date guidance in the rapidly changing field of melanoma treatment, guideline developers have to provide regular updates without compromises of quality. We performed a systematic search in guideline databases, Medline and Embase to identify guidelines on CM. The methodological quality of the identified guidelines was independently assessed by five reviewers using the instruments “Appraisal of Guidelines for Research and Evaluation” (AGREE II) and “Recommendation EXcellence” (AGREE-REX). We performed descriptive analysis, explored subgroup differences using the Kruskal–Wallis (H) test and examined the relationship between distinct domains and items of the instruments with Spearman’s correlation. Six guidelines by consortia from Australia, France, Germany, Scotland, Spain and the United States of America were included. The German guideline fulfilled 71%–98% of criteria in AGREE II and 78%–96% for AGREE-REX, obtaining the highest scores. Deficiencies in the domains of “applicability” and “values and preferences” were observed in all guidelines. The German and Spanish guidelines significantly differed from each other in most of the domains. The domains “applicability” and “values and preferences” were identified as methodological weaknesses requiring careful revision and improvement in the future.

## 1. Introduction

The incidence of primary cutaneous melanoma (CM) continues to increase steadily each year [1,2]. In 2018, more than 287,000 new cases were diagnosed globally, ranking melanoma among the 20 most commonly diagnosed cancer entities in the world [3]. Surgical treatment is usually curative following early detection of the disease [3,4]. However, melanoma rapidly becomes life-threatening once it metastasizes [4], although considerable progress has been made following the introduction of targeted therapies using BRAF and MEK inhibitors and novel immunotherapies [5,6].

The management of melanoma is subject to country-specific health care conditions. Evidence-based clinical guidelines are important tools for clinicians, assisting them in finding the best therapy option for their patients, as well as providing guidance for diagnostic procedures and surveillance. They include statements and recommendations for the optimization and standardization of patient care, which are developed based on a systematic literature review of evidence and an evaluation of the benefits and harms of various care options [1]. A multitude of novel treatment options and drug combinations has recently been approved for the treatment of CM in the adjuvant setting and advanced disease within the last few years, and the field is evolving rapidly [2]. Thus, guidelines have to be updated frequently to provide up-to-date information, and may lag behind current developments.

Numerous evaluation tools have been developed for assessing the methodological quality of guidelines [3,4,5]. The most widely used and validated tool is the “Appraisal of Guidelines for Research and Evaluation (AGREE) II”, which was also recommended by WHO [6,7]. In 2009, AGREE II was published as a revised version of the original AGREE instrument, which was released eight years before. It includes 23 items which are grouped into six domains and two additional overall assessment items [7]. Recently, an AGREE-II-complementing evaluation tool named “AGREE-REX: Recommendation EXcellence” has been introduced [8]. It was newly developed to evaluate the clinical credibility and implementability of practice guideline recommendations, and was intended to be a strategy to inform their development and reporting. In this article, we used the evaluation tools AGREE II and AGREE-REX to critically evaluate the methodological quality of current guidelines for melanoma treatment, which were identified in a systematic literature search.

## 2. Results

### 2.1. Guideline Identification

**Figure 1:**
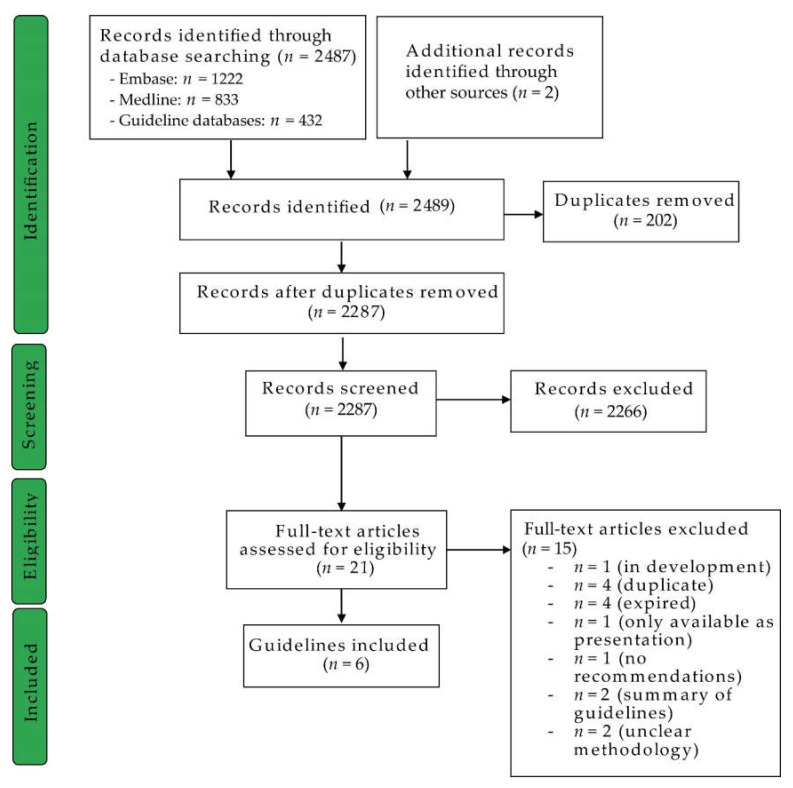
Flow chart illustrating the process of guideline identification according to the PRISMA guidelines [32]. Searched guideline databases: Arbeitsgemeinschaft der Wissenschaftlichen Medizinischen Fachgesellschaften, Ärztliches Zentrum für Qualität in der Medizin, Arzneimittelkommission der deutschen Ärzteschaft; National Institute for Health and Care Excellence, Guidelines International Network, Agency for Healthcare Research and Quality, Scottish Intercollegiate Guidelines Network, Dutch Guidelines Oncoline.

Our initial database search identified 2489 records (Figure 1). Twenty-one records were included in the full-text review after title and abstract screening and removal of duplicates. Of these guidelines, four were dismissed, as they had already expired at the time of our search [9,10,11,12], one was in development [13] and another one was only available as a presentation [14]. Furthermore, we identified four more duplicates [15,16,17,18] and two records were excluded due to unclear methodological approaches [19,20]. One record did not present any recommendations [21], and two records provided a summary of existing guidelines [22,23,24]. Hence, six relevant guidelines published between 2017 and 2019 by consortia of Australia, France, Germany, Scotland, Spain and the United States of America were included in the appraisal (Table 1) [25,26,27,28,29,30]. Altogether, the overall interrater agreement was estimated as fair for the two tools used for this evaluation (Fleiss’ Kappa = 0.209, 95% CI: 0.190–0.227) [31].

### 2.2. AGREE II

#### 2.2.1. Scope and Purpose

**Figure 2:**
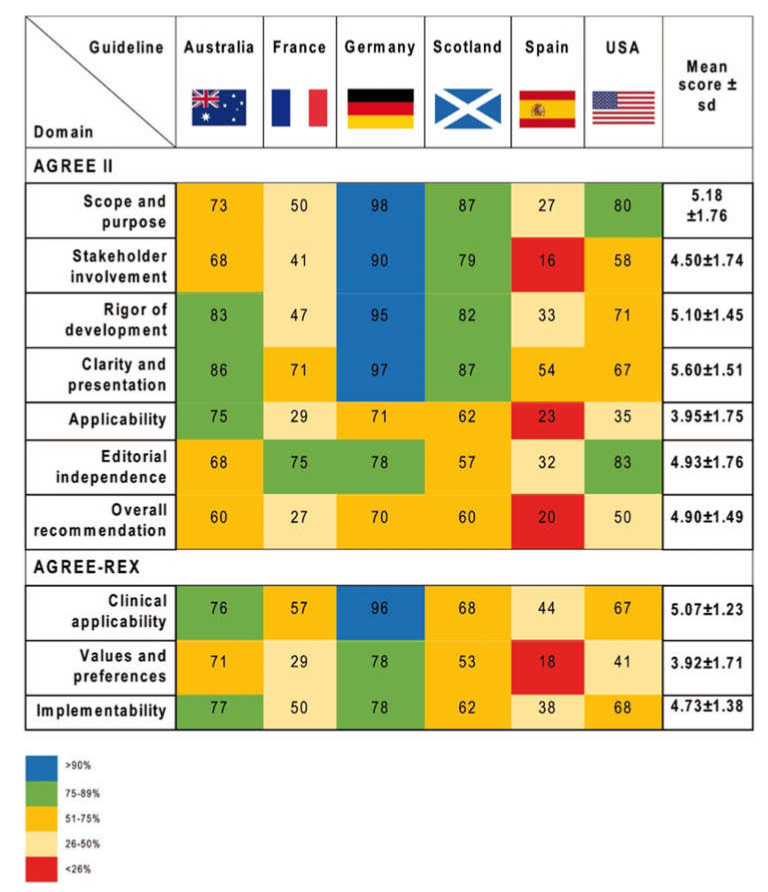
Overview of the final “Appraisal of Guidelines for Research and Evaluation” (AGREE II) and “Recommendation EXcellence” (AGREE-REX) scores. The heat map shows the average score of each domain for each cutaneous melanoma (CM) guideline evaluated by five independent reviewers.

**Figure 3:**
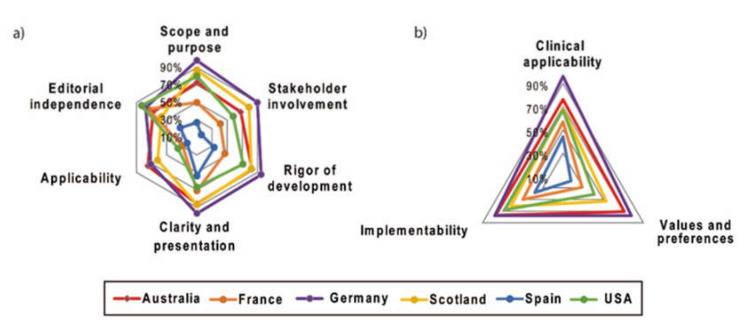
Networks comparing the six different guidelines regarding the different domains of (**a**) AGREE II and (**b**) AGREE-REX.

In this domain, the main objectives of the guidelines and their intended target population were evaluated. Overall, an average score of 5.18 was achieved (± 1.76) (Figure 2 and Figure 3a, and Table S1). The German guideline achieved the highest score and fulfilled 98% of the criteria, whereas the guideline from Spain achieved only 27%. The German and the Spanish (*p* = 0.001), as well as the German and the French guideline (*p* = 0.035), significantly differed from each other in this domain.

#### 2.2.2. Stakeholder Involvement

This domain assesses whether appropriate stakeholders were involved in the developing process, and whether the views of the intended guideline users are represented. A mean score of 4.50 (± 1.74) was achieved. The fulfilled values ranged from 16% for the Spanish guideline to 90% for the German guideline. The Scottish guideline was significantly different from the Spanish guideline (*p* = 0.019) in this domain. Additionally, the Spanish and the German guideline significantly differed from each other (*p* = 0.001).

#### 2.2.3. Rigor of Development

This domain focuses on the methodology and assesses whether the evidence for the guideline was gathered in a systematic and transparent search. The mean score was 5.10 (± 1.45). Besides, the German guideline showed the best methodological quality in this domain (95% of items fulfilled), followed by the guidelines from Australia and Scotland with 83% and 82% of items fulfilled, respectively. The other guidelines varied from 33% to 71%. Additionally, the German guideline was significantly different from the Spanish (*p* = 0.001) and the French (*p* = 0.009) ones.

#### 2.2.4. Clarity and Presentation

This domain assesses the format and presentation of guidelines and evaluates whether the key recommendations can be easily identified, and whether the recommendations are described specifically and unambiguously. The mean score of this domain was high (5.60 ± 1.51); the German guideline was rated as the best guideline (97% of fulfilled criteria), followed by the Scottish (87%) and Australian (86%) guideline.

#### 2.2.5. Application

The domain “application” focuses on the relevant processes for a successful guideline implementation, including facilitators, barriers and the provision of additional material. It also evaluates whether monitoring and/or auditing criteria are described. Interestingly, this domain had the lowest overall mean score of all domains (3.95 ± 1.75). The Australian guideline was rated as the best compared to the remaining guidelines (75%), while the Spanish guideline achieved the lowest score (23%).

#### 2.2.6. Editorial Independence

This domain investigates funding and the competing interests of the experts who were involved in guideline development. The mean score of this domain was 4.93 (± 1.76). The US guideline fulfilled 83% of the criteria, whereas the Spanish guideline demonstrated 32% of fulfilled editorial independence items.

#### 2.2.7. Overall Assessment

The overall quality and whether the guideline can be recommended for use is evaluated in this final domain. The overall score was 4.90 (± 1.49) and the fulfilled criteria ranged from 20% (Spanish guideline) to 70% (German guideline). Therefore, the guidelines from Australia, Germany, Scotland and the US would be uniformly recommended for use in practice, whereas the two guidelines from Spain and France were recommended with modifications only, or rated not to be recommended at all by the five reviewers.

### 2.3. AGREE-REX

#### 2.3.1. Clinical Applicability

This domain assesses whether the guideline was developed based on evidence, and also evaluates whether the recommendations are applicable for the intended target users setting. The mean score was 5.07 ± 1.23, and the German guideline was rated as the best clinically applicable guideline with 96%, whereas the guideline from Spain was rated lowest with 44% (Figure 2 and Figure 3b, and Table S2). The German guideline significantly differed from the Spanish (*p* = 0.002) and the French guidelines in this criterion (*p* = 0.025).

#### 2.3.2. Values and Preferences

This domain evaluates the relative importance that the target guideline developers, users, policy/decision-makers and patients attach to outcomes of interest. Overall, the mean score was 3.92 ± 1.71. The guidelines from Australia and Germany achieved high values with 71% and 78% of items fulfilled. The Spanish guideline yielded the lowest score with 18% of items fulfilled. Significant differences were found between the Spanish and the Australian guidelines (*p* = 0.046) as well as for the Spanish and German guidelines (*p* = 0.010).

#### 2.3.3. Implementability

This domain evaluates whether the recommendations are suitable for the patients, population or the health care systems which they were developed for. It investigates whether the guideline recommendations align with the implementation goals of the guideline, as well as whether the guidelines include advice or tools and resources to facilitate the implementation of the recommendations, since these are easier to adopt in practice. Again, the German and Australian guidelines achieved the highest results with 77% and 78%, respectively. The mean score was 4.73 ± 1.38. Besides, a significant difference was identified between the Spanish and the German guidelines (*p* = 0.010).

### 2.4. Correlations of the AGREE II and AGREE-REX Domains

Most of the AGREE II domains were significantly correlated with each other (Table 2). The domains “scope and purpose” and “stakeholder involvement” were significantly positively correlated with all other domains. “Scope and purpose” showed a high positive correlation with the domains “stakeholder involvement” (*r* = 0.92), “overall assessment” (*r* = 0.85) and “rigor of development” (*r* = 0.81). There was also a strong, positive correlation between the domain “stakeholder involvement” and the domains “rigor of development” (*r* = 0.89), “clinical applicability” (*r* = 0.85) and “overall assessment” (*r* = 0.84). In addition, “clinical applicability” was positively associated with “rigor of development” (*r* = 0.86). Notably, all AGREE-REX domains were positively and statistically significantly correlated with each other.

## 3. Discussion

Altogether, the German guideline was evaluated best with both instruments. It appeared to strictly adhere to the AGREE domains and described the guideline developing process in detail. This may be explained by the set of rules that the Association of the Scientific Medical Societies in Germany (AWMF) has established for the creation of guidelines, and to which guideline developers have to adhere. Besides, thorough and continuous methodological support is provided by the German Cancer Society and AWMF, which are both involved in the development of cancer guidelines in Germany. A similarly strict framework is provided by the SIGN consortium responsible for the development of the Scottish guidelines that also achieved high scores in this evaluation [28,33]. The Australian and US guidelines were also developed based on a predefined set of rules for guideline development [25,30]. However, it remains unclear whether the French and Spanish consortia had to adhere to specific rules during their respective development processes [26,29]. Importantly, we did not perform an analysis of the content of the included guidelines, but instead focused exclusively on their methodological quality [34].

The field is rapidly evolving, as new treatments and treatment combinations are steadily on the rise, and also since more analyses from clinical studies and long-term follow-up are available [35,36,37]. In this context, guideline developers are facing two major challenges. First, they are confronted with a constantly growing knowledge base. In melanoma, this challenge predominantly affects the treatment of advanced disease, while other domains such as epidemiology, diagnostics and surveillance are by far less subject to fluctuation and the dynamics of the underlying body of evidence. Second, the quality standards for guideline development are becoming increasingly stringent, and the steps of literature research, appraisal of evidence and consensus processes increasingly become more complex and formalized as advancements in the standards, methods and tools of evidence-based medicine are being achieved. This inevitably results in a conflict between the challenge of keeping guidelines up to date and their compliance with rigorous quality standards. The period of guideline validity is usually 2–6 years, while early updates of the entire guideline and amendments of specific recommendations are possible. Guidelines are recommended for updates as soon as novel impactful evidence is available for a given recommendation. However, to update an existing guideline in its entirety is time-consuming and labor-intensive. Here, the living guideline model can facilitate updates of individual recommendations in a flexible and targeted manner [38,39]. This model requires prompt and sensitive identification of substantial, i.e., practice-changing, evidence from newly published studies, and a rapid update and dissemination by the guideline panels and stakeholders. Current practice guidelines of melanoma increasingly struggle with the pace of developments. We propose that a living guideline format would help to better cope with this issue rather than regular updates at fixed intervals without compromising the methodological quality. Since none of the instruments assesses whether guidelines are up to date, this category should be included in future modifications of the instruments. As the German guideline was evaluated best among all studies that were included in the quality assessment, it may serve as a template for other consortia for the development of future guidelines. Additionally, tools like an online-comment function or a function allowing to pose questions for a living model need to be urgently included.

This study has several limitations. The fact that all reviewers of this study are from Germany might have influenced and positively biased the evaluation of the German guideline, which had achieved the best results among all six guidelines. However, none of the evaluators was part of the guideline committee. Besides, we only included guidelines that had been available in English or in the German language. Hence, the assessment may be biased.

## 4. Materials and Methods

### 4.1. Eligibility Criteria

For our appraisal, we included national and international guidelines with a focus on the treatment of CM in the English or German languages, as the field in melanoma treatment has been rapidly changing over the previous years. Besides, guideline chapters on treatment, especially covering palliative and (neo-) adjuvant approaches, are often dynamically modified and updated regularly for melanoma. The guidelines had to be published within the last 3 years (i.e., 2016–2019), as we aimed to appraise the most recent and up-to-date guidelines while simultaneously accounting for a time lag for the implementation of newly approved interventions, which began in 2011 with the approval of ipilimumab by the Food and Drug Administration. Furthermore, the period of validity of guidelines is usually 2–6 years. These aspects suggest that a time frame from 2016–2019 appeared appropriate. Furthermore, we restricted our search to guidelines which were developed based on a systematic literature assessment and a structured consensus process, i.e., evidence- and consensus-based guidelines. Guidelines covering only a specific aspect of melanoma were excluded.

### 4.2. Search Strategy and Guideline Selection

We systematically searched for eligible guidelines in several guideline databases, including multidisciplinary and subject-specific guideline providers (Table S3), Medline and Embase (both via Ovid) until 20 December 2019. The detailed search strategy is available in Table S4. The initial search results were screened and double hits were removed. Afterwards, two authors (M.V.H., T.S.) independently screened the titles and abstracts of the remaining records. Full texts of potentially relevant guidelines were obtained. After this, the guidelines were checked if they fulfilled our predefined eligibility criteria.

### 4.3. Data Extraction and Rating of the Guidelines

Two authors (T.S., M.V.H.) independently collected relevant information on each guideline, including title, national authority/author and country of origin, publication date, methodological approach and its scope. Five independent reviewers assessed the methodological quality of each guideline identified in the search using the evaluation tools AGREE II and AGREE-REX as recently described by the authors [40].

### 4.4. Analysis

All domain scores were calculated according to the AGREE II and AGREE-REX instructions [7,8]. The total scores describe the percentage of the maximum score that can be achieved for each domain. Thus, they can range from 0% (worst) to 100% as the best achievable score. SPSS (version 24, IBM Corporation, Armonk, NY, USA) was used for all statistical analyses. Mean (± standard deviation, SD) or median and interquartile ranges (IQR) were calculated as part of the descriptive analyses, and subgroup differences were explored with the Kruskal–Wallis (H) test. Spearman’s correlation was used to examine the relationship between the distinct domains, and *p*-values < 0.05 were considered as statistically significant. The interrater agreement of the five reviewers was determined using Fleiss’ Kappa [31].

## 5. Conclusions

Altogether, this analysis demonstrates that only a few of the currently available melanoma guidelines are of high methodological quality. The published guideline from Germany showed the best rating, suggesting that it may be used as a template for other guideline projects. The domains “applicability” and “values and preferences” had achieved the lowest scores in our evaluation, which should be taken into consideration in future guideline development processes. A domain that needs to be included in future evaluations is whether these guidelines are up-to-date.

## Figures and Tables

**Table 1:** Overview of the six identified guidelines on cutaneous melanoma.

Title	Year	National Society and/or Authors
Clinical practice guidelines for the diagnosis and management of melanoma [25]	2019	Cancer Council Australia
French updated recommendations in Stage I to III melanoma treatment and management [26]	2017	Guillot et al., (France)
Diagnostik, Therapie und Nachsorge des Melanoms [27]	2019	AWMF, DKG, DKH (Germany)
Cutaneous melanoma [28]	2017	Scottish Intercollegiate Guidelines Network (SIGN)
SEOM clinical guideline for the management of malignant melanoma [29]	2017	Berrocal et al. (Spain)
Guidelines of care for the management of primary cutaneous melanoma [30]	2019	Swetter et al. (USA)

**Table 2:** Correlations among the distinct AGREE II and AGREE-REX domains; *: *p* < 0.05, **: *p* < 0.01.

*r* < 0.29		AGREE II							AGREE-REX		
*r* ≥ 0.3–0.49											
*r* ≥ 0.5–0.69											
*r* ≥ 0.7–0.89											
*r* ≥ 0.9											
Domain		Scope and Purpose	Stakeholder Involvement	Rigor of Development	Clarity of Presentation	Applicability	Editorial Independence	Overall Assessment	Clinical Applicability	Values and Preferences	Implementability
**AGREE II**	**Scope and Purpose**	1.000	0.917 **	0.811 **	0.716 **	0.569 **	0.422 *	0.845 **	0.770 **	0.555 **	0.638 **
	**Stakeholder Involvement**		1.000	0.890 **	0.684 **	0.639 **	0.427 *	0.839 **	0.845 **	0.584 **	0.589 **
	**Rigor of Development**			1.000	0.536 **	0.680 **	0.326	0.798 **	0.858 **	0.660 **	0.571 **
	**Clarity of Presentation**				1.000	0.418 *	0.457 *	0.739 **	0.624 **	0.299	0.505 **
	**Applicability**					1.000	0.021	0.587 **	0.785 **	0.735 **	0.585 **
	**Editorial Independence**						1.000	0.375 *	0.276	−0.050	0.127
	**Overall Assessment**							1.000	0.768 **	0.581 **	0.617 **
**AGREE-REX**	**Clinical applicability**								1.000	0.730 **	0.686 **
	**Values and preferences**									1.000	0.663 **
	**Implementability**										1.000

## Supplementary Materials

The following are available online at https://www.mdpi.com/2072-6694/12/6/1613/s1, Table S1: Overview of AGREE II mean scores (± standard deviation) of the six CM guidelines assessed by the five independent reviewers on a scale ranging from 1 (lowest quality) to 7 (highest quality), Table S2: Overview of AGREE-REX mean scores (± standard deviation) of the six CM guidelines assessed by five independent reviewers on a scale ranging from 1 (lowest quality) to 7 (highest quality), Table S3: Overview of the guideline databases searched for “cutaneous melanoma”, Table S4: Overview of the search strategy in Medline and Embase via Ovid.
