# Supplementary material for: The Quality of Practice Guidelines for Melanoma: A Methodologic Appraisal with the AGREE II and AGREE-REX Instruments
Source: Cancers (Basel). 2020 Jun 18;12(6):1613. doi: 10.3390/cancers12061613 (PMC7352745; doi:10.3390/cancers12061613)
Supplement: Supplementary file 1 [file cancers-12-01613-s001.pdf]

Supplementary Materials

The Quality of Practice Guidelines for Melanoma: A Methodologic Appraisal with the AGREE II and AGREE-REX Instruments

Theresa Steeb, Anja Wessely, Konstantin Drexler, Martin Salzmann, Frédéric Toussaint, Lucie Heinzerling, Markus Reinholz, Carola Berking and Markus V. Heppt

Table S1. Overview of AGREE II mean scores (± standard deviation) of the 6 CM guidelines assessed by 5 independent reviewers on a scale ranging from 1 (lowest quality) to 7 (highest quality).

Domain/item	Australia	France	Germany	Scotland	Spain	USA
**Scope and Purpose**						
1 Description overall objectives	6.00 ± 0.71	3.80 ± 1.79	6.80 ± 0.45	6.40 ± 0.55	2.40 ± 1.67	5.00 ± 1.58
2 Description health questions	7.00 ± 0.00	4.40 ± 1.82	6.80 ± 0.45	6.40 ± 0.55	2.80 ± 1.30	6.20 ± 1.30
3 Description of patient population at focus	3.20 ± 1.80	3.80 ± 2.12	7.00 ± 0.00	5.80 ± 1.79	2.60 ± 1.82	6.20 ± 1.79
Overall percentage of scope and purpose	73%	50%	98%	87%	27%	80%
**Stakeholder Involvement**						
4 Relevant groups included in guideline group	6.80 ± 0.45	4.60 ± 0.89	6.00 ± 1.73	6.00 ± 1.00	3.00 ± 1.41	5.00 ± 2.12
5 Patient preferences considered	5.00 ± 1.00	2.40 ± 1.52	6.20 ± 0.45	5.60 ± 1.14	1.20 ± 0.45	4.40 ± 1.67
6 Target users defined	3.40 ± 2.10	3.20 ± 1.64	7.00 ± 0.00	5.60 ± 1.95	1.60 ± 0.55	4.00 ± 1.23
Overall percentage of stakeholder involvement	68%	41%	90%	79%	16%	58%
**Rigor of Development**						
7 Systematic literature research	6.40 ± 0.89	5.20 ± 0.45	7.00 ± 0.00	6.80 ± 0.45	2.60 ± 1.34	6.40 ± 0.89
8 Description of literature selection criteria	5.20 ± 2.12	4.60 ± 1.52	6.80 ± 0.45	5.20 ± 1.30	2.20 ± 0.45	6.40 ± 0.89
9 Strengths and limitations of evidence	5.40 ± 0.89	5.20 ± 0.45	6.60 ± 0.55	5.40 ± 1.34	4.20 ± 1.79	4.80 ± 1.10
10 Description methods for formulating recommendations	6.40 ± 0.89	4.00 ± 2.12	7.00 ± 0.00	5.40 ± 1.14	2.40 ± 1.67	4.80 ± 1.30
11 Side-effect, risks considered in recommendations	5.40 ± 1.34	2.60 ± 2.07	6.20 ± 0.84	6.20 ± 0.45	3.40 ± 1.52	5.80 ± 0.45
12 Link between evidence and recommendations	6.80 ± 0.45	4.20 ± 2.95	7.00 ± 0.00	6.40 ± 1.34	5.40 ± 1.52	6.00 ± 1.23
13 External review prior to publication	5.40 ± 1.14	2.80 ± 1.10	6.20 ± 1.30	6.40 ± 1.34	2.40 ± 1.14	4.40 ± 1.67
14 Procedure for updating provided	6.80 ± 0.45	1.80 ± 0.45	6.60 ± 0.55	5.60 ± 2.61	1.00 ± 0.00	3.60 ± 2.07
Overall percentage of methodology	83%	47%	95%	82%	33%	71%
**Clarity and Presentation**						
15 Recommendation specific and unanimous	5.80 ± 1.10	4.80 ± 2.17	6.80 ± 0.45	6.00 ± 1.73	4.60 ± 1.95	4.80 ± 1.92
16 Clear presentation of different treatment options	5.80 ± 0.84	4.00 ± 2.00	6.60 ± 0.55	6.00 ± 1.23	4.80 ± 1.79	5.20 ± 2.05
17 Key recommendations easily identifiable	6.60 ± 0.55	7.00 ± 0.00	7.00 ± 0.00	6.60 ± 0.89	3.40 ± 2.41	5.00 ± 2.74
18 Tools for application available	5.40 ± 2.51	2.40 ± 1.95	3.60 ± 2.07	3.20 ± 1.92	2.00 ± 1.41	2.60 ± 2.30
Overall percentage of clarity and presentation	86%	71%	97%	87%	54%	67%
**Applicability**						
19 Discussion of organizational facilitators and barriers	6.40 ± 0.55	2.40 ± 1.95	6.20 ± 1.10	5.40 ± 1.52	2.00 ± 1.41	2.60 ± 2.30
20 Consideration of costs of guideline implementation	5.00 ± 2.55	2.40 ± 1.94	5.00 ± 1.41	5.20 ± 1.92	2.00 ± 1.41	3.00 ± 1.52
21 Inclusion of monitoring/auditing criteria	5.20 ± 2.05	3.80 ± 1.64	6.20 ± 0.84	5.00 ± 1.58	3.60 ± 1.34	4.20 ± 1.10
Overall percentage of applicability	75%	29%	71%	62%	23%	35%
**Editorial Independence**						
22 Editorial independence from funding body	5.00 ± 1.00	6.20 ± 1.30	5.60 ± 1.14	4.00 ± 2.00	2.40 ± 1.95	6.20 ± 1.30
23 Reporting of conflicts of interests	5.20 ± 2.49	4.80 ± 2.39	5.80 ± 2.68	4.80 ± 2.28	3.40 ± 2.41	5.80 ± 2.68

Overall percentage of editorial independence	68%	75%	78%	57%	32%	83%
**Total Score**	5.80 ± 0.45	3.40 ± 0.89	6.60 ± 0.55	5.80 ± 0.45	2.80 ± 0.45	5.00 ± 0.71
**Overall Assessment**	60%	27%	70%	60%	20%	50%

Table S2. Overview of AGREE-REX mean scores (± standard deviation) of the 6 CM guidelines assessed by 5 independent reviewers on a scale ranging from 1 (lowest quality) to 7 (highest quality).

Item	Australia	France	Germany	Scotland	Spain	USA	Overall assessment
**Clinical Applicability**							
Evidence	6.60 ± 0.55	4.00 ± 1.23	6.80 ± 0.45	5.40 ± 1.95	4.80 ± 2.17	5.40 ± 1.34	5.50 ± 1.64
To users	5.20 ± 1.10	5.40 ± 0.55	6.80 ± 0.45	5.00 ± 1.23	3.20 ± 1.10	5.00 ± 1.58	5.10 ± 1.45
To patients and populations	4.80 ± 0.84	3.80 ± 1.48	6.60 ± 0.55	4.80 ± 1.79	3.00 ± 1.58	4.60 ± 2.19	4.60 ± 1.77
Overall percentage	76%	57%	96%	68%	44%	67%	
**Values and Preferences**							
Of target users	5.00 ± 1.41	3.40 ± 1.67	6.00 ± 1.41	5.40 ± 0.89	3.20 ± 1.79	4.60 ± 1.34	4.60 ± 1,67
Of patients and populations	5.40 ± 0.55	2.20 ± 1.64	6.20 ± 0.45	4.80 ± 1.64	1.40 ± 0.89	4.00 ± 1.58	4.00 ± 2.07
Of policy and decision-makers	5.00 ± 2.35	3.40 ± 1.95	5.20 ± 2.49	3.20 ± 2.28	1.60 ± 0.55	2.20 ± 1.64	3.43 ± 2.25
Of guideline developer	5.60 ± 0.89	2.00 ± 1.23	5.40 ± 1.52	3.40 ± 1.52	2.20 ± 1.63	3.00 ± 1.23	3.60 ± 2.01
Overall percentage	71%	29%	78%	53%	18%	41%	
**Implementability**							
Purpose	5.60 ± 0.89	4.20 ± 1.30	5.80 ± 1.10	4.80 ± 1.64	4.00 ± 1.23	5.20 ± 0.45	4.93 ± 1.26
Local application and adoption	5.60 ± 0.55	3.80 ± 1.48	5.60 ± 1.14	4.60 ± 2.07	2.60 ± 0.89	5.00 ± 1.41	4.53 ± 1.63
Overall percentage	77%	50%	78%	62%	38%	68%	

Table S3. Overview of the guideline databases searched for "cutaneous melanoma".

Guideline Database (Last Update: 20 December 2019)	Initial Hits
Arbeitsgemeinschaft der Wissenschaftlichen Medizinischen Fachgesellschaften: AWMF (https://www.awmf.org/leitlinien/leitlinien-suche.html)	27
Ärztliches Zentrum für Qualität in der Medizin: ÄZQ (leitlinien.de)	0
Arzneimittelkommission der deutschen Ärzteschaft (AkdÄ) (http://www.akdae.de)	66
National Institute for Health and Care Excellence: NICE (guidance.nice.org.uk/CG/Published)	20
Guidelines International Network: GIN (g-i-n.net)	22
Agency for Healthcare Research and Quality: NGC (guidelines.gov/)	277
Scottish Intercollegiate Guidelines Network: SIGN (sign.ac.uk/)	18
Dutch Guidelines (oncoline.nl/index.php?language=en)	2
Cross references	2

Table S4. Overview of the search strategy in Medline and Embase via Ovid.

**Search Query in Medline (Ovid MEDLINE (R) and Epub Ahead of Print, In-Process & Other Non-Indexed Citations, Daily and Versions (R) 1946 to December 20, 2019; *n* = 833 hits)**
1. practice guideline.mp. or exp Practice Guideline/
2. health planning guideline.mp. or exp Health Planning Guidelines/
3. guideline*.m_titl.
4. clinical protocol.mp or exp. Clinical Protocols/
5. guidance.m_titl.
6. guidance.mp.
7. care pathway.mp.
8. exp Evidence-Based Medicine/
8. consensus development.mp. or exp Consensus/
10. exp Guideline/ or guideline.mp
11. 1 or 2 or 3 or 4 or 5 or 6 or 7 or 8 or 9 or 10
12. exp Melanoma/ or melanoma.mp
13. 11 and 12
13. limit 12 to (english or german)
14. limit 13 to yr = "2016-Current"
**Search Query in Embase (Embase 1974 to 2019 December 20 *n* = 1,222 hits)**
1. practice guideline.mp. or exp practice guideline/

2. health planning guidelines.mp. or exp health care planning/
3. guideline.mp.
4. care pathway.mp.
5. consensus development.mp. or exp consensus development/
6. exp consensus/ or consensus.mp
7. 1 or 2 or 3 or 4 or 5 or 6
8. exp melanoma/ or exp cutaneous melanoma/
9. 7 and 8
10. limit 9 to (english or german)
11. limit 10 to yr = "2016-Current"

© 2020 by the authors. Licensee MDPI, Basel, Switzerland. This article is an open access article distributed under the terms and conditions of the Creative Commons Attribution (CC BY) license (http://creativecommons.org/licenses/by/4.0/).
